# Accessibility of State and Territory Public Health Department Website Information on COVID-19 Outpatient Treatments in the US

**DOI:** 10.1001/jamanetworkopen.2023.0186

**Published:** 2023-02-21

**Authors:** Cody Eckert, Mechelle Sanders, Rhea Bharadwaj, Kevin A. Fiscella

**Affiliations:** 1 University of Rochester Medical Center, Rochester, New York

## Abstract

This cross-sectional study assesses the reading level and accessibility of information on COVID-19 treatments posted on US public health websites for states, territories, and Washington, DC.

## Introduction

Life-saving oral and injectable antiviral therapeutics are available to treat non-hospitalized patients with a high risk of COVID-19 infection. Yet uptake of treatments is suboptimal and inequitable.^1^ Public health websites are vital information sources for patients seeking timely COVID-19 treatment.^2^ We assessed the readability and accessibility of these websites, particularly for people with low literacy or limited English language proficiency.

## Methods

This cross-sectional study of US public health websites for states, territories, and Washington, DC, was deemed exempt from review and the requirement for informed consent according to the Common Rule because it was considered non–human participant research. We adhered to the STROBE reporting guideline for cross-sectional studies.

All authors reviewed and coded websites for accessibility (eMethods in Supplement 1) according to 8 categories created by consensus: (1) ease of navigation to the web page containing treatment options; (2) oral and injectable options for treatment; (3) eligibility requirements for treatment; (4) how and where to obtain treatment; (5) treatment costs and availability for uninsured patients; (6) text languages other than English, including American Sign Language (ASL); (7) telephone support, including mention of Telecommunications Devices for the Deaf, and (8) ASL access. All authors independently rated website, with 2 raters assessing each website from June to July 2022 using the following grading system for accessibility: 0, not met; 1, partially met; and 2, fully met. We resolved differences through discussion of textual evidence and consensus.

We assessed readability ease for websites with treatment information using Flesch reading ease scores (0-100), with higher scores reflecting easier reading level. We assessed κ statistics for inter-rater reliability for accessibility categories and the mean or median accessibility score for each website. We conducted multivariable logistic regression analysis to assess independent associations of governors’ political affiliation, population size, and public health spending per person with scoring above the median accessibility score. A 2-sided *P* < .05 was considered statistically significant. We used Stata 12.0 (StataCorp, Inc) for statistical analyses.

## Results

The sample included a total of 56 public health websites for 50 states, 5 territories, and Washington, DC. There was substantial agreement (70%) for accessibility ratings (κ= 0.582; SE, 0.05; *P* < .001). The mean (SD) and median (range) ease of readability scores were 46.03 (12.95)and 43.70 (12.40-93.20), respectively, indicating difficult readability. One state and 2 territories did not provide COVID-19 treatment information, were not assessed for readability, and were rated 0 for accessibility (Table).^3,4^ The readability scores ranged from 12.4 to 93.2, corresponding to very difficult to read (ie, college graduate or International Standard Classification of Education [ISCED-6]) to very easy to read (ie, fifth-grade reading level or ISCED-1). Only 3 websites (5%) had a readability score of 70 or higher (ie, fairly easy to read at or below a 7th-grade reading level). Vermont, Minnesota, and Oregon had the highest mean (SD) accessibility ratings (1.75 [0.46], 1.88 [0.35], and 1.88 [0.35], respectively). Only 2 states, Oklahoma and Vermont, received high ratings for ASL support.

**Table.** Accessibility and Readability of State, Territory, and Washington, DC, Public Health Department Website Information for COVID-19 Treatment in the US

Location	Mean (SD) accessibility score^a^	Readability score^b^	Reading ease	Approximate US reading grade (ISCED level)^c^	Governor’s political affiliation^d^	Population size, quartile^e^	Public health spending per person, $^f^
Alabama	0.88 (0.64)	57.6	Fairly difficult	10-12th (3)	Republican	2	52.35
Alaska	1.13 (0.83)	54.9	Fairly difficult	10-12th (3)	Republican	4	90.17
American Samoa	0	Not rated	Not rated	Not rated	Independent	4	NA
Arizona	0.25 (0.46)	41.0	Difficult	College (5)	Republican	1	15.32
Arkansas	1.25 (0.89)	38.7	Difficult	College (5)	Republican	3	42.94
California	1.13 (0.64)	38.0	Difficult	College (5)	Democrat	1	78.47
Colorado	1.38 (0.74)	32.0	Difficult	College (5)	Democrat	2	51.48
Connecticut	0.88 (0.64)	38.2	Difficult	College (5)	Democrat	2	37.46
Delaware	1.13 (0.83)	49.5	Difficult	College (5)	Democrat	4	NA
Washington, DC	0.63 (0.52)	60.4	Standard	8-9th (2)	Democrat	4	370.56
Florida	1.25 (0.89)	35.1	Difficult	College (5)	Republican	1	19.53
Georgia	1.13 (0.83)	43.7	Difficult	College (5)	Republican	1	30.82
Guam	1.13 (0.99)	48.2	Difficult	College (5)	Democrat	4	NA
Hawaii	1.13 (0.83)	56.4	Fairly difficult	10-12th (3)	Democrat	3	121.95
Idaho	1.13 (0.83)	41.2	Difficult	College (5)	Republican	3	80.74
Illinois	1.00 (0.76)	38.5	Difficult	College (5)	Democrat	1	33.85
Indiana	1.25 (0.89)	40.0	Difficult	College (5)	Republican	2	15.34
Iowa	0.88 (0.99)	33.8	Difficult	College (5)	Republican	3	36.91
Kansas	0.75 (0.71)	66.8	Standard	8-9th (2)	Democrat	3	NA
Kentucky	1.50 (0.76)	53.6	Fairly difficult	10-12th (3)	Democrat	2	29.64
Louisiana	1.13 (0.64)	28.0	Very difficult	College graduate (6)	Democrat	2	41.21
Maine	1.38 (0.74)	72.5	Fairly easy	7th (2)	Democrat	3	44.34
Maryland	1.63 (0.74)	49.3	Difficult	College (5)	Republican	2	108.77
Massachusetts	1.63 (0.52)	57.2	Fairly difficult	10-12th (3)	Republican	2	104.32
Michigan	1.50 (0.53)	42.8	Difficult	College (5)	Democrat	1	19.78
Minnesota	1.88 (0.35)	45.0	Difficult	College (5)	Democrat	2	77.33
Mississippi	1.50 (0.76)	41.6	Difficult	College (5)	Republican	3	15.73
Missouri	1.25 (0.71)	38.6	Difficult	College (5)	Republican	2	6.54
Montana	1.25 (0.89)	32.1	Difficult	College (5)	Republican	4	18.33
Nebraska	0	Not rated	Not rated	Not rated	Republican	3	40.33
Nevada	1.00 (0.93)	43.6	Difficult	College (5)	Democrat	3	13.52
New Hampshire	0.75 (0.89)	43.7	Difficult	College (5)	Republican	3	24.72
New Jersey	1.50 (0.76)	52.8	Fairly difficult	10-12th (3)	Democrat	1	32.44
New Mexico	1.38 (0.74)	44.8	Difficult	College (5)	Democrat	3	159.25
New York	1.13 (0.83)	59.4	Fairly difficult	10-12th (3)	Democrat	1	92.44
North Carolina	1.50 (0.76)	35.6	Difficult	College (5)	Democrat	1	14.8
North Dakota	1.25 (0.89)	37.2	Difficult	College (5)	Republican	4	61.18
Northern Mariana	0	Not rated	Not rated	Not rated	Republican	4	NA
Ohio	1.00 (0.93)	49.8	Difficult	College (5)	Republican	1	24
Oklahoma	1.38 (0.74)	12.4	Very difficult	College graduate (6)	Republican	2	89.47
Oregon	1.88 (0.35)	45.8	Difficult	College (5)	Democrat	2	35.51
Pennsylvania	1.38 (0.92)	43.6	Difficult	College (5)	Democrat	1	15.75
Puerto Rico	1.38 (0.92)	30.9	Difficult	College (5)	Democrat	3	NA
Rhode Island	1.63 (0.74)	37.1	Difficult	College (5)	Democrat	4	NA
South Carolina	1.25 (0.71)	33.0	Difficult	College (5)	Republican	2	27.15
South Dakota	1.25 (0.71)	93.2	Very easy	6th (2)	Republican	4	37.06
Tennessee	0.88 (0.83)	72.6	Fairly easy	7th (2)	Republican	2	70.35
Texas	1.25 (0.89)	50.3	Fairly difficult	10-12th (3)	Republican	1	17.91
Utah	1.00 (0.93)	47.5	Difficult	College (5)	Republican	3	NA
Vermont	1.75 (0.46)	57.5	Fairly difficult	10-12th (3)	Democrat	4	56.18
Virgin Islands	0.88 (0.64)	50.2	Fairly difficult	10-12th (3)	Democrat	4	NA
Virginia	1.50 (0.76)	54.4	Fairly difficult	10-12th (3)	Republican	1	41.98
Washington	1.25 (0.71)	43.8	Difficult	College (5)	Democrat	1	89
West Virginia	1.00 (0.76)	51.0	Fairly difficult	10-12th (3)	Republican	3	NA
Wisconsin	1.50 (0.76)	42.4	Difficult	College (5)	Democrat	2	17.47
Wyoming	0.75 (0.89)	32.1	Difficult	College (5)	Republican	4	27.23

Abbreviation: ISCED, International Standard Classification for Education.

^a^ Accessibility was based on the mean of scores for 8 categories, with each score ranging from 0 to 2.

^b^ Readability score was measured using Flesch reading ease scores (range, 0-100, with higher scores indicating an easier reading level).

^c^ ISCED ranges from 0 to 8 and approximates US educational level.

^d^ Political affiliation of the governor’s political party in 2022 based on an internet search of each state’s governor.

^e^ Quartile 1, ≥7.26 million; quartile 2, 3.99 to 6.99 million; quartile 3, 1.39 to 3.62 million; quartile 4, ≤1.38 million.^3^

^f^ Public health spending in US dollars per capita 2021.^4^

Generally, accessibility ratings were lowest for deaf access (0.20), payment information (0.73), and telephone support (1.03). Few websites had text in non-English languages and relied on Google Translate. Democratic affiliation was associated with increased odds (odd ratio, 7.59; 95% CI, 1.70-33.97; *P* = .008) of scoring above the median accessibility score. Other variables were not statistically significant.

## Discussion

We found that COVID-19 treatment information on US public health websites was poorly accessible, particularly for people with low literacy or limited English language proficiency, with worse accessibility for states and territories with Republican governors. These findings are consistent with those of other studies.^5,6^ We found that website navigation was easier when COVID-19 treatment information was displayed on the website rather than providing hyperlinks to external federal government websites. Study limitations include no testing of telephone lines and dynamic updates to websites.

Findings of this cross-sectional study underscore the poor accessibility and readability of COVID-19 treatment information on US public health websites, which may contribute to inequities in access to life-saving treatment. The results suggest the need for national guidelines on accessibility and readability for public health websites.

## Notes

Supplement 1. **eMethods.** Criteria for Rating Public Health Department Websites

Supplement 2.
Data Sharing Statement
