# Error in the Table and Results

**DOI:** 10.1001/jamanetworkopen.2023.31664

**Published:** 2023-08-22

In the research letter titled “Accessibility of State and Territory Public Health Department Website Information on COVID-19 Outpatient Treatments in the US”^1^ published on February 21, 2023, the political affiliation of the governor of Vermont was incorrectly identified as Democrat rather than Republican. In addition, the odds ratio, 95% CI, and *P* value reported in the penultimate sentence of the Results section were incorrect. The sentence should be the following: “Democratic affiliation was associated with increased odds (odds ratio, 4.97; 95% CI, 1.22-20.20; *P* = .03) of scoring above the median accessibility score.” The article has been corrected.
