# Supplementary material for: Accessibility of State and Territory Public Health Department Website Information on COVID-19 Outpatient Treatments in the US
Source: JAMA Netw Open. 2023 Feb 21;6(2):e230186. doi: 10.1001/jamanetworkopen.2023.0186 (PMC9945065; doi:10.1001/jamanetworkopen.2023.0186)
Supplement: Supplement 1. — eMethods. Criteria for Rating Public Health Department Websites [file jamanetwopen-e230186-s001.pdf]

Supplemental Online Content

Eckert C, Sanders M, Bharadwaj R, Fiscella KA. Accessibility of state and territory public health department website information on COVID-19 outpatient treatments in the US. *JAMA Netw Open*. 2023;6(2):e230186. doi:10.1001/jamanetworkopen.2023.0186

eMethods. Criteria for Rating Public Health Department Websites

This supplemental material has been provided by the authors to give readers additional information about their work.

© 2023 Eckert C et al. *JAMA Network Open.*

eMethods. Criteria for Rating Public Health Department Websites

Navigation of Website
0= No information about treatment options on the COVID-19 webpage (or if none the home page).
1= Treatment option link to HHS (federal government) website(s) the COVID-19 webpage (or if none the home page)
2= Treatment options specifically mentioned on the COVID-19 webpage (or if none the home page)

Options for COVID-19 Outpatient Treatment
0= None were mentioned, and no links were provided
1= Options available through links
2= Both oral and injectable options mentioned on website

Requirements for Treatment
0= No requirements for treatment mentioned
1= Mentions requirement for a positive COVID-19 test and window period
2= Mentions a positive COVID test and window period and high-risk conditions and/or age

Information on How to Obtain COVID-19 Treatment
0= No information
1= information provided on location/phone numbers of providers/sites directly or through links
2= information provided on location/phone numbers of providers/sites directly and on pharmacies including the specific drug and supply directly or through links

Payment for COVID-19 visits and Treatment
0= No mention that treatment is free
1= Indicates free treatment is available
2= Indicates that free treatment is available and where to obtain it

Written Language Available
0= No non-English languages listed
1= Spanish written materials or Google Translate
2= Information in multiple written languages and Google Translate

Phone Support on the Website
0= No phone support listed
1= Phone support available including for non-English speakers
2= Phone support available including non-English speaks and TTY/TTD

Deaf Access on the Website
0= No ASL videos/links

© 2023 Eckert C et al. *JAMA Network Open.*

1= ASL video available or links to other websites with video
2= ASL video regarding treatment options posted on the original website

Readability
Flesch readability ease
https://www.webfx.com/tools/read-able/

ASL=American Sign Language
COVID-19= coronavirus disease 2019 from the SARS-CoV-2 virus
Flesch readability ease score is calculated using the formula:
Readability Ease = 206.835 – (1.015 x Average Sentence Length) – (84.6 x Average Syllables per Word)

TTY/TTD= A teletypewriter/ Telecommunications Device for the Deaf are communication devices used by people who are deaf, hard-of-hearing, or have severe speech impairment.

© 2023 Eckert C et al. *JAMA Network Open.*
