# Supplementary material for: Accessibility of State and Territory Public Health Department Website Information on COVID-19 Outpatient Treatments in the US
Source: JAMA Netw Open. 2023 Feb 21;6(2):e230186. doi: 10.1001/jamanetworkopen.2023.0186 (PMC9945065; doi:10.1001/jamanetworkopen.2023.0186)
Supplement: Supplement 2. — Data Sharing Statement [file jamanetwopen-e230186-s002.pdf]

Data Sharing Statement

Eckert. Accessibility of State and Territory Public Health Department Website Information on COVID-19 Outpatient Treatments in the US. *JAMA Netw Open*. Published February 21, 2023. doi:10.1001/jamanetworkopen.2023.0186

Data
Data available: Yes
Data types: Data (not involving human participants)
How to access data: The supplementary files include the criteria used for each of the seven accessibility criteria. There is no need for a data dictionary for this very simple dataset. The actual scoring for each of the seven categories is available upon request to: kevin_fiscella@urmc.rochester.edu.
When available: With publication

Supporting Documents
Document types: None

Additional Information
Who can access the data: These data will be shared with any researcher requesting them. No IRB approval is needed.
Types of analyses: Any purpose.
Mechanisms of data availability: Any request.
Any additional restrictions: Attribution to our team if our scores are used.
